# Case Report: Single-port laparoscopic total gastrectomy for gastric cancer in patient with situs inversus totalis

**DOI:** 10.3389/fonc.2023.1094053

**Published:** 2023-01-18

**Authors:** In Young Lee, Danbi Lee, Chang Min Lee

**Affiliations:** ^1^ Department of Surgery, Korea University College of Medicine, Seoul, Republic of Korea; ^2^ Department of Surgery, Korea University Medical Center Ansan Hospital, Ansan, Gyeonggi-do, Republic of Korea

**Keywords:** gastric cancer, laparoscopic, single-port, situs inversus totalis, total gastrectomy

## Abstract

Situs inversus totalis (SIT) is a rare anatomical condition, where all the viscera appear in its reverse position. Although minimally invasive surgery has evolved to achieve totally laparoscopic gastrectomy for gastric cancer patients with SIT, it is difficult to perform lymphadenectomy in such a transposed anatomical condition. Recently, we performed a single-port laparoscopic total gastrectomy (SPTG) for gastric cancer in a patient with SIT. No postoperative complications or dietary problems were observed. Based on this experience, we are to design a safe strategy to perform D2 lymphadenectomy during SPTG in patients with SIT.

## 1 Introduction

Situs inversus totalis (SIT) is a rare anatomical condition, in which all the thoracic and abdominal viscera appear in the reverse position. In case of gastric cancer patient with situs inversus, surgical procedures are technically demanding because most surgeons are not familiar with the transposed anatomy. In particular, the transposition of the vascular structures causes considerable confusion throughout the operation, because gastric cancer surgery includes lymphadenectomies around named vessels.

Ever since Yamaguchi et al. (1) first reported laparoscopy-assisted distal gastrectomy (LADG) for a gastric cancer patient with SIT in 2003, several surgeons have performed laparoscopic surgeries in patients with SIT. Although minimally invasive surgery (MIS) has evolved in the last two decades to achieve totally laparoscopic distal gastrectomy and totally laparoscopic total gastrectomy for gastric cancer in patients with SIT (2, 3), it is still difficult to accomplish lymph node dissection (LND) in such a transposed anatomical condition.

One significant issue in performing laparoscopic surgery in a patient with SIT is how to position the surgeons (including the operator, assistants, and scopist) and monitors in the operation room. Owing to the unfamiliarity with the SIT condition, surgeries performed using both right- and left-sided approaches cause considerable discomfort during lymphadenectomy (3). Several solutions have been introduced to manage this issue. Kigasawa et al. (4) used a two-monitor method, in which one monitor showed direct intra-abdominal images and the other showed mirrored images during LADG in a patient with SIT; however, they described that there still were difficulties in LND. Another remarkable solution was the introduction of a robotic surgical system. Kim et al. (5) described that the technical difficulties in patients with SIT were somewhat resolved with the aid of a robotic surgical system; the surgeon could perform the surgery using both right- and left-sided approaches freely, as the positions of the instruments could be freely changed. Furthermore, the operator feels it is less awkward to switch between the dominant and non-dominant hands in the transposed anatomies, since the right- or left-handedness is considerably filtered during robotic surgery.

However, we contemplated that the robotic surgical system is not appropriate in dealing with unexpected situations, including massive bleeding or unintended organ injury in unfamiliar anatomical conditions; therefore, we adopted the laparoscopic approach in MIS for patients with SIT.

Recently, we performed a single-port laparoscopic total gastrectomy in a patient with gastric cancer and SIT. In this case report, we describe the technical details of the surgical procedure and postoperative outcomes.

## 2 Case report

A 79-year-old woman was referred to our hospital after a clinical diagnosis of gastric cancer. The patient presented without any symptoms. Gastrofiberscopy showed an elevated mucosal lesion with the longest diameter of approximately 4 cm, located in the posterior wall of the gastric high body. Although high-grade dysplasia was diagnosed on pathological examination, the gastroenterologist who performed the gastrofiberscopy described the impression as early gastric cancer; the preoperative gastrofiberscopic findings, including the fusion and retraction of the gastric folds, corresponded to the features of submucosal invasion in patients with gastric cancer. Computed tomography revealed SIT; however, it did not indicate any distant metastases or peritoneal carcinomatosis. Her body mass index was 20.5 kg/m^2^. The patient had no history of abdominal surgery.

We planned a single-port laparoscopic surgery for this patient. Total gastrectomy, rather than proximal gastrectomy, was considered because the tumor showed an ambiguous border and had a wide shape. The details of our procedure were as follows.

### 2.1 Preparative procedures

The patient was placed on a surgical table with both legs abducted. After general anesthesia was induced, the surgical table was adjusted to create a reverse Trendelenburg position. The operator stood between the patient’s legs, and the scopist was positioned on the patient’s right side.

A commercial 4-lumen single-port trocar (Gloveport^®^, Nelis, Bucheon, Korea) was inserted through a trans-umbilical incision using Hasson’s method (6). After a pneumoperitoneum was created with carbon dioxide at a pressure of 15 mmHg, the falciform ligament and the left lobe of the liver were raised toward the cephalad direction by combined suture retraction (7). We could not find any evidence of incurable findings in the laparoscopic view.

### 2.2 LND for gastric cancer (Video S1)

**Figure 1:**
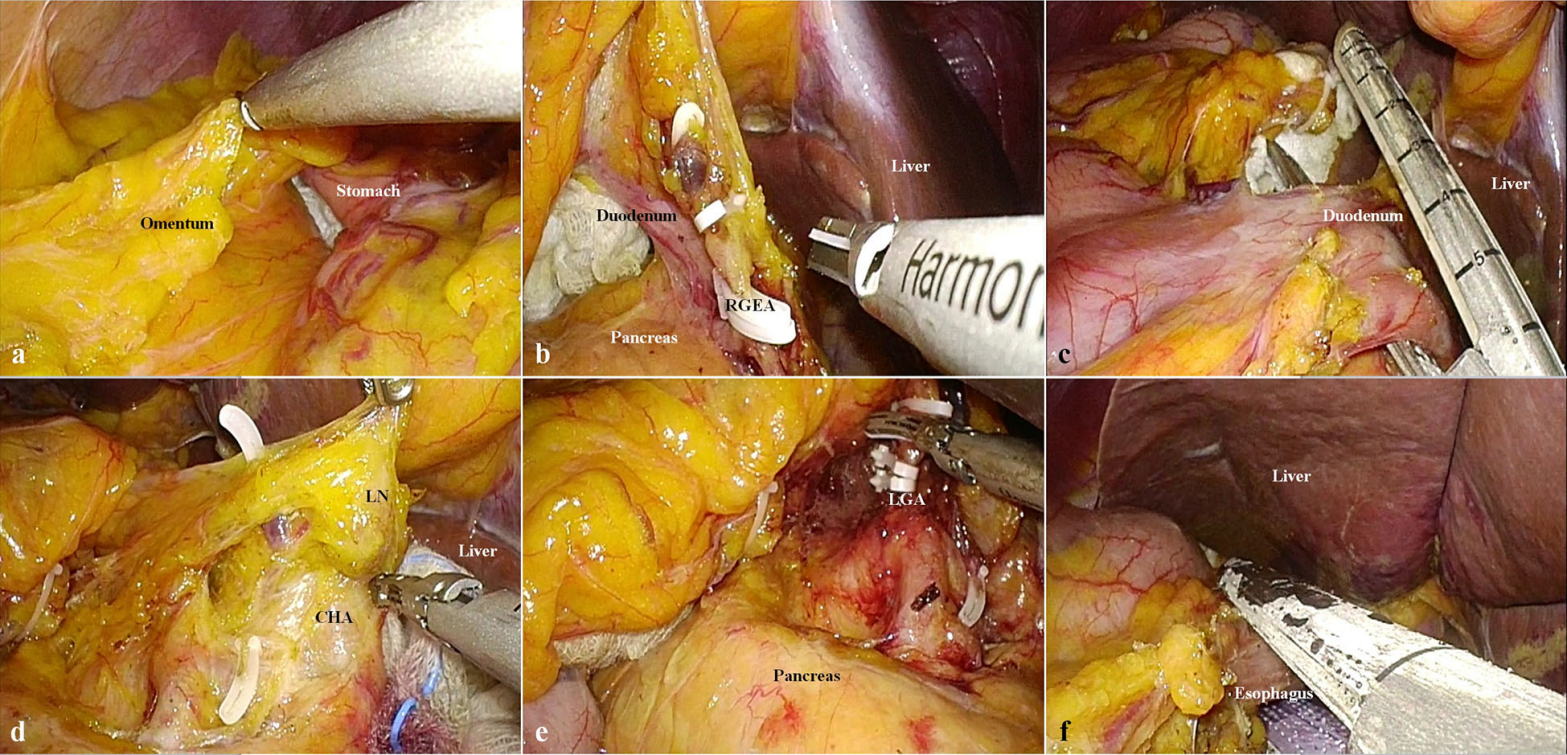
Steps of lymph node dissection. **(A)** Omentectomy toward the left gastroepiploic vessels. **(B)** Ligation and division of the left gastroepiploic vessels (RGEA, right gastroepiploic artery). **(C)** Division of the duodenum using a laparoscopic linear stapler. **(D)** Lymphadenectomy of lymph node station No. 8a (CHA, common hepatic artery; LN, lymph node). **(E)** Ligation and division of the left gastric artery (LGA, left gastric artery). **(F)** Division of the esophagus using a laparoscopic linear stapler.

D2 lymphadenectomy was performed according to the Japanese Gastric Cancer Treatment Guidelines 2014 (ver. 4) (8). Harmonic Ace 7 (Ethicon Endo-Surgery Inc., Cincinnati, OH, USA) was used to facilitate LND. The details of our procedure were as follows:

i) Partial omentectomy (**Figure 1A**) began with the division of the greater omentum more than 4 cm from the gastroepiploic arcade to include lymph node (LN) station No. 4d. The left gastroepiploic artery was ligated and divided to dissect the LN station No. 4sb.
ii) The greater omentum was divided to mobilize the distal stomach. The right gastroepiploic vessels were ligated and divided to include LN station No. 6 (**Figure 1B**).
iii) Supra-duodenal dissection was performed to create a window toward the gastro-hepatic vascular structures. Subsequently, the right gastric vessels were ligated and divided.
iv) The duodenum was divided using the Signia™ Stapling System (Tri-Staple™ 60-mm tan cartridge; Medtronic, Minneapolis, MN, U.S.A.) (**Figure 1C**).
v) LN station No. 8a was dissected until the common hepatic artery was completely exposed (**Figure 1D**).
vi) With the stomach pushed toward the right side, suprapancreatic LND was performed to clear LN stations No. 7, 9, and 11p. As LN station No. 7 was dissected, the left gastric artery and coronary vein were ligated and divided (**Figure 1E**).
vii) After the right and left crus were exposed to dissect LN station No. 9, the abdominal esophagus was cleared. Subsequently, the esophagus was divided using the Signia™ Stapling System (Medtronic) (**Figure 1F**). Lymphadenectomy was performed by dividing the short gastric vessels, and the LN station No. 10 was omitted.

### 2.3 Reconstruction (Video S2)

**Figure 2:**
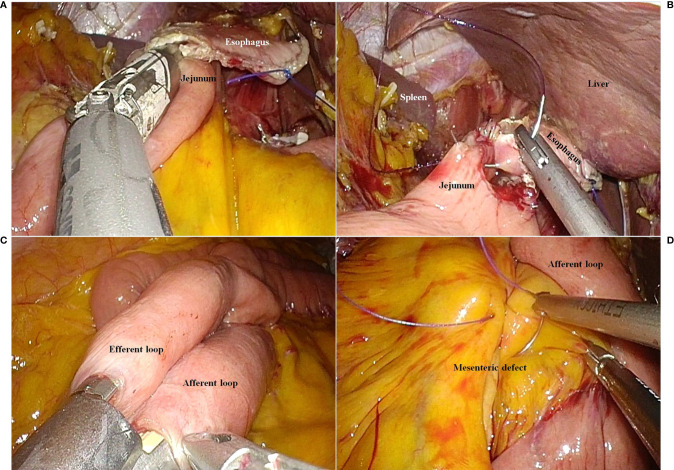
The reconstruction procedure. **(A)** Esophagojejunostomy using a laparoscopic linear stapler. **(B)** Closure of the common entry hole using a barbed suture material. **(C)** Jejunojejunostomy using a laparoscopic linear stapler. **(D)** Closure of the mesenteric defect due to jejunojejunostomy.

On the point that the jejunal limb could reach the esophageal stump, the jejunum was divided using the Signia™ Stapling System (Medtronic). To restore continuity of the digestive tract, an esophagojejunostomy was performed using the Signia™ Stapling System (Medtronic) (**Figure 2A**). The common hole due to the stapled esophagojejunostomy was closed using a 3-0 absorbable barbed suture (**Figure 2B**). To connect the biliopancreatic limb to the digestive tract, a jejunojejunostomy was performed using the Signia™ Stapling System (Medtronic) (**Figure 2C**). We closed the common hole due to the stapled jejunojejunostomy using a 3-0 absorbable barbed suture. The jejunojejunostomy-associated mesenteric defect was closed using a 3-0 non-absorbable barbed suture (**Figure 2D**).

### 2.4 Drainage

**Figure 3:**
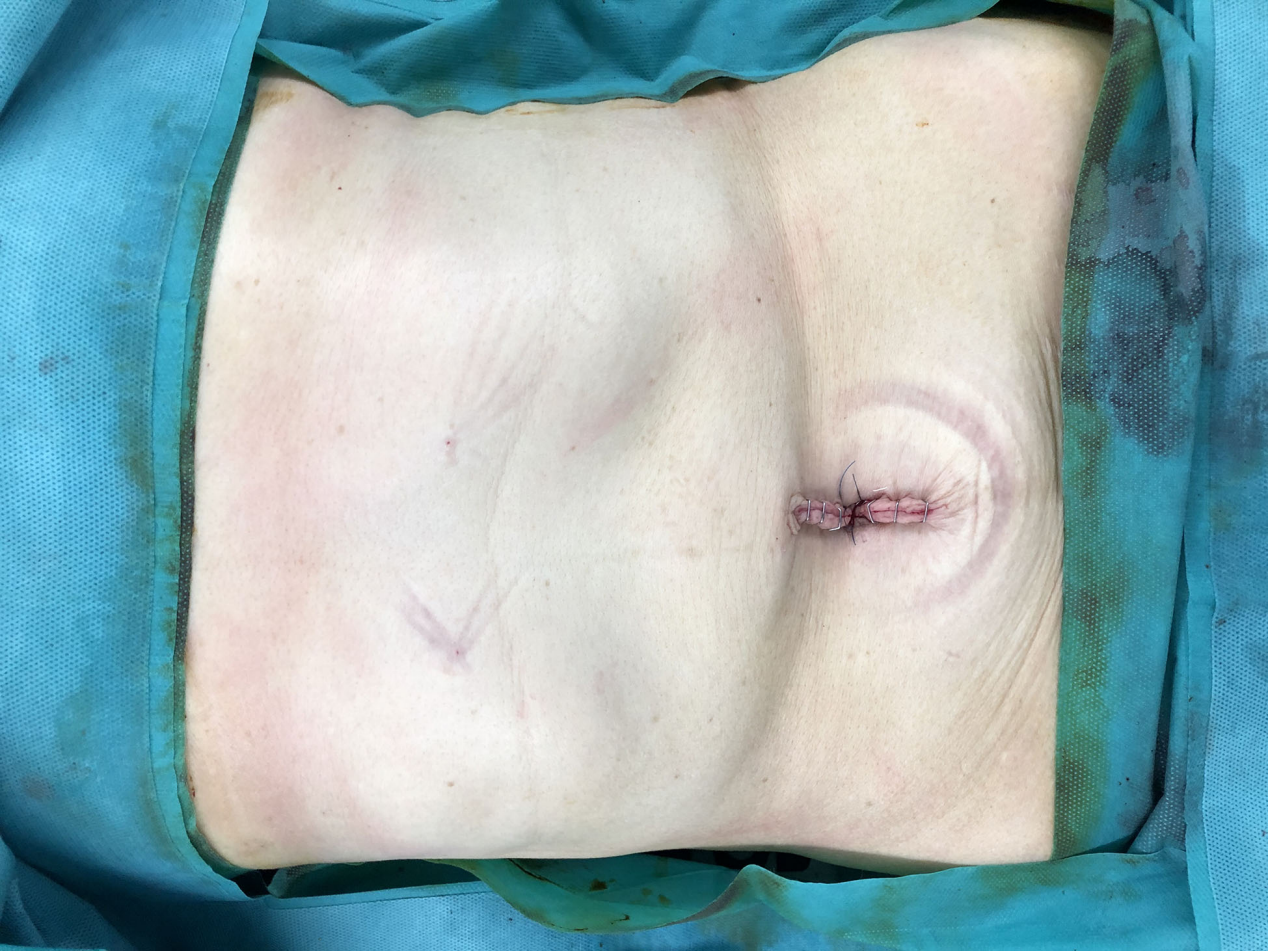
The postoperative wound. There was only one trans-umbilical wound in the abdomen.

No drainage tube was established (**Figure 3**).

### 2.5 Clinical outcomes

The total operative time was 269 min. The patient started drinking water on postoperative day 5. A semi-bland diet was provided on postoperative day 7. The patient was discharged on postoperative day 14 without any adverse events. The final pathology report revealed that the tumor had penetrated the gastric serosa. No LN metastasis among the 41 LNs that had been dissected was observed (pT4aN0, stage IIB, according to the American Joint Committee on Cancer, 8^th^ ed.) (9).

The patient was followed up on postoperative day 22. No evidence of wound infections or dietary problems was found. Since then, the patient has received S-1 monotherapy as adjuvant chemotherapy, and a total of eight cycles have been completed without dose reduction. We did not find any evidence of recurrence 24 months after surgery.

## 3 Discussion

Laparoscopic surgery for gastric cancer is difficult to perform in patients with SIT. The first problem surgeons encounter is determining how to establish a camera and working ports in the patient’s abdomen. Regardless of the position preferred by the operator during laparoscopic gastrectomy, all procedures need to be performed using different approaches in patients with SIT. If surgeons choose the same position that they usually adopt for gastric cancer surgery (right-sided approach), they will have to deal with the abdominal organs in different locations. If surgeons try to accomplish the same procedures on the side opposite to the position they are familiar with (left-side approach), they will need to change the role of each hand. Therefore, regardless of the approach selected, the surgeon will be unfamiliar with the surgical procedure to be performed in patients with SIT (3).

In this patient with SIT, we performed a single-port laparoscopic gastrectomy (SPLG) to overcome unfamiliarity due to the transposed anatomy. Although SPLG involves technically demanding procedures, several strategies associated with the single-port laparoscopic approach for patients with SIT are available.

Moreover, we could alleviate the unfamiliarity caused by the right- or left-sided approach in patients with SIT. In the original SPLG procedure, the surgeon stood between the patient’s legs. Likewise, in the current case, the operator was positioned between the patient’s legs. We expected that this position might provide the operator with a similar vector toward the organs as when performing SPLG in patients with normal anatomy. In addition, for patients in whom all organs were inverted, it might induce less confusion (than right- or left-sided approach) to manipulate the organs in the median position. In this patient with SIT, “median positioned” approach causes a few unique situations. First, during omentectomy toward the left gastroepiploic vessels, the left-handed grasper could not raise the stomach (the cephalad traction of the omentum and stomach is performed using a left-handed instrument in patients with the normal anatomy). Therefore, gauzes had to be inserted to acquire adequate space between the omentectomy line and the stomach (**Figure 1A**). Second, during lymphadenectomy of LN station No. 6, there was less collision between the instruments than during SPLG in patients with normal anatomy. Generally, the duodenum is retracted upwards using a left hand-controlled grasper, while the right hand-controlled energy device is used for LND. In this patient, the grasper and energy device rarely needed to intercross intracorporeally, since the duodenum was located on the left side. Third, during the dissection of LN station No. 8a, we had to pay special attention to apprehend the inverted anatomy, including the named arteries arising from the celiac trunk (**Figure 1E**).

Another strategy for the SIT condition is the use of special instruments associated with the SPLG. At the initial period of establishing SPLG, we had used the prebent grasper (Olympus Medical Systems Corp., Tokyo, Japan) to avoid collision between the instruments and scope in the multi-channel trocar rim (10, 11). More recently, we have replaced the prebent grasper (Olympus Medical Systems Corp.) with a multi-degree of freedom (DOF) articulating instrument, ArtiSential (LivsMed, Seongnam, Korea) (12). The main mechanism for avoiding collisions during single-port surgery is to reflect the axis of the instrument. However, because we could not predict the direction toward which the instrument should be reflected in the SIT condition, we expected the multi-DOF articulating instrument might be more advantageous than the prebent grasper. ArtiSential can be freely reflected in any direction, whereas we the prebent grasper need to be re-assembled extracorporeally in order to change the direction of reflection. This feature enabled us to adapt to the transposed anatomy in the SIT patient.

Nevertheless, there remains some concerns regarding the long-term outcome, although this patient had no postoperative complications and recurrence for 2 years after the operation. In particular, the final pathology report showed that the pathologic stage was IIB (pT4aN0), which was so discrepant from the preoperative diagnosis. The preoperative endoscopic biopsy suggested that the gastric tumor was a high-grade dysplasia. Although we performed a total gastrectomy in preparation for the possibility of gastric cancer, we did not accomplish a D2 lymphadenectomy. Most of all, the first concerning point is related to the individual outcome of this patient. Despite the pathologic diagnosis of stage IIB, we cannot ignore the possibility of ‘stage migration,’ in which the stage might be underestimated due to the deficient LND. Unfortunately, this stage (IIB) was considered in the process of adopting the chemotherapeutic regimen for adjuvant therapy; we were concerned about whether S-1 monotherapy was an appropriate choice for this patient. Although there is no first-level evidence that S-1 monotherapy is inferior to the XELOX regimen, XELOX is preferred in patients with stage III gastric cancer at our institute.

Another concern correlates with the legitimacy of our surgical strategy. In this case, because we did not consider the possibility of advanced gastric cancer (AGC), D1+ lymphadenectomy was carried out. Regarding this issue, we should consider how to extend surgical radicality in patients with SIT. Although D2 lymphadenectomy has been achieved in laparoscopic surgery for AGC, it is necessary to design an upgraded strategy for future cases of SIT. Although we suggested that SPLG was a surgical solution in patients with SIT, we did not attempt D2 lymphadenectomy in a patient who had been preoperatively diagnosed with high-grade dysplasia. However, in cases of encountering patients with preoperatively-confirmed AGC, single-port laparoscopic approach might be an infeasible strategy for D2 LND in patients with SIT. Likewise, while some surgeons who perform SPLG have the expertise to perform D2 lymphadenectomy in patients with normal anatomy (13), experience is limited for patients with SIT.

**Figure 4:**
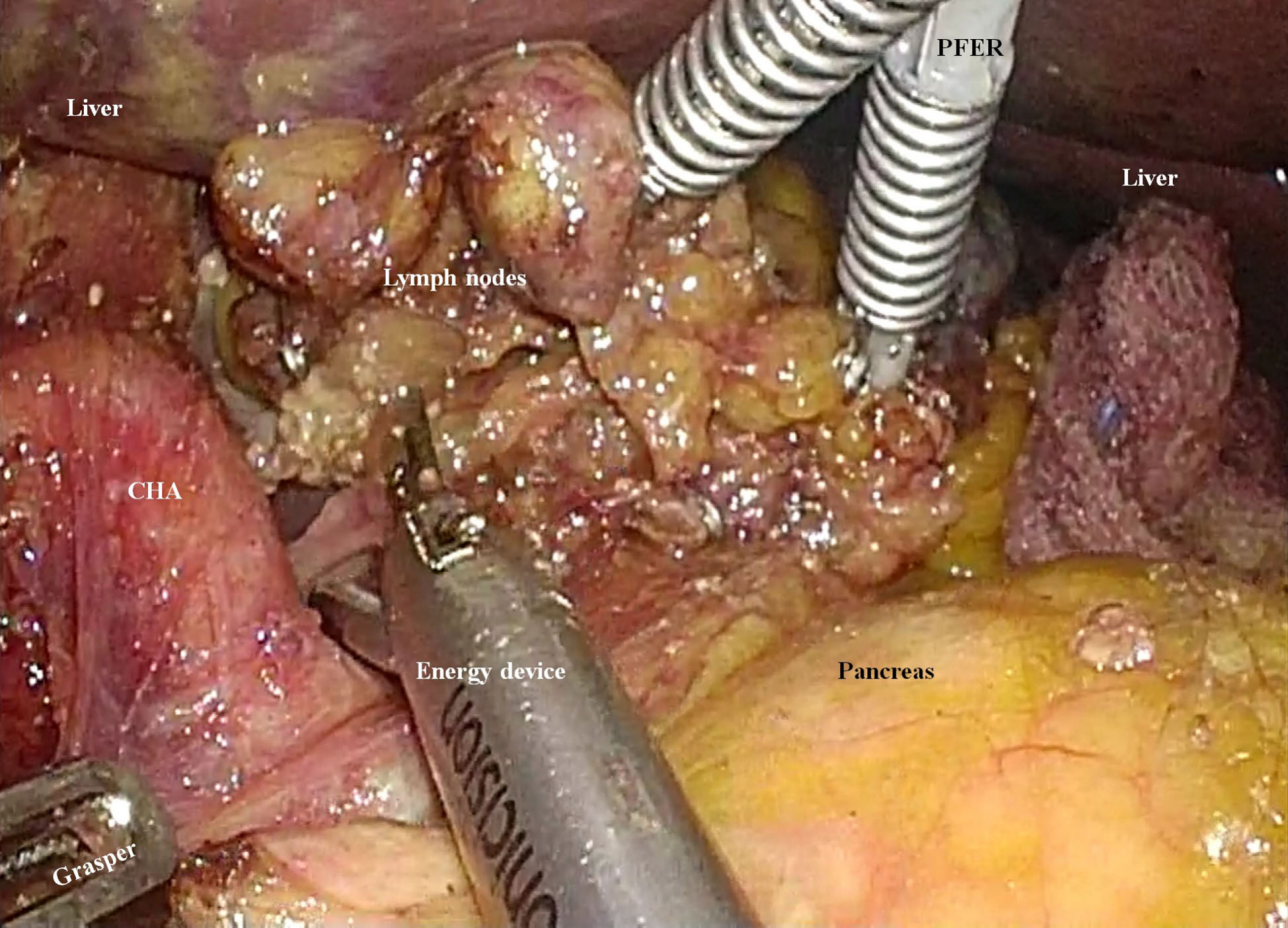
An instrument for enhancing the surgical field during single-port laparoscopic gastrectomy. The EndoGrab™ Port-Free Endocavity Retractor (Virtual Ports, Misgav, Israel) provides stable counter-traction without making any additional incisions (CHA, common hepatic artery; PFER, Port-Free Endocavity Retractor).

Therefore, if we were to encounter patients with preoperatively-confirmed AGC in the future, the following strategies should be considered for D2 lymphadenectomy. First, we should not stick to the “unaided” type SPLG, in which a surgeon performs all the surgical procedures without any assistance (11, 14–16). In the recent days, we introduced EndoGrab™ Port-Free Endocavity Retractor (Virtual Ports, Misgav, Israel) in laparoscopic gastrectomy. With this instrument, in the left hand, the surgeon can manipulate the other tissues or organs to enhance the surgical view (**Figure 4**) (17). However, such an instrument was not used when we performed SPLG for this patient with SIT. After performing a surgical treatment for this patient, we have been accustomed to using the EndoGrab™ Port-Free Endocavity Retractor (Virtual Ports) in single-port surgery for other patients as well. This device is expected to simulate the condition of the assistant surgeon adding counter-traction to achieve D2 lymphadenectomy during SPLG.

Second, it is not necessary to limit the length and number of incisions when performing radical surgery in patients with AGC. One reason for adopting SPLG in the patient with SIT was that the operator could be centrally positioned to avoid deviation to either side of the patient. However, if we were to encounter a patient with preoperatively-confirmed AGC and SIT, we would not limit ourselves to a single trans-umbilical incision with a length of 2.5 cm. In such cases, although we would still pursue the central position between the patient’s legs, a longer incision or additional working ports would be considered to achieve stable outcomes in the short- and long-term.

In summary, to our best knowledge, this was the first experience of performing a single-port laparoscopic total gastrectomy in a gastric cancer patient with SIT. According to our expertise regarding SPLG, single-port laparoscopic approach was adopted to overcome the unfamiliar condition due to SIT. The additional strategies should be considered to perform D2 lymphadenectomy in the future SIT cases.

## Data availability statement

The original contributions presented in the study are included in the article/**Supplementary Material**. Further inquiries can be directed to the corresponding author.

## Ethics statement

Written informed consent was obtained from the individual for the publication of any potentially identifiable images or data included in this article.

## Author contributions

IL write the manuscript. DL collected the data and interpreted the data. CL designed the main concept of this study and verified all the contents of the study and manuscript. All authors contributed to the article and approved the submitted version.
